# NMR resonance assignments for a docking domain pair with an attached thiolation domain from the PAX peptide-producing NRPS from *Xenorhabdus cabanillasii*

**DOI:** 10.1007/s12104-021-10010-1

**Published:** 2021-03-05

**Authors:** Jonas Watzel, Sepas Sarawi, Elke Duchardt-Ferner, Helge B. Bode, Jens Wöhnert

**Affiliations:** 1 Molecular Biotechnology, Institute of Molecular Biosciences, Goethe University Frankfurt, 60438 Frankfurt am Main, Germany; 2 Institute of Molecular Biosciences and Center for Biomolecular Magnetic Resonance (BMRZ), Goethe University Frankfurt, 60438 Frankfurt am Main, Germany; 3 Buchmann Institute for Molecular Life Sciences (BMLS), Goethe University Frankfurt, 60438 Frankfurt am Main, Germany; 4 Senckenberg Gesellschaft Für Naturforschung, 60325 Frankfurt am Main, Germany; 5 Department of Natural Products in Organismic Interactions, Max-Planck-Institute for Terrestrial Microbiology, 35043 Marburg, Germany

**Keywords:** NMR assignments, Peptide-antimicrobial-*Xenorhabdus* (PAX) peptide, Non-ribosomal peptide synthetase (NRPS), Docking domain, Thiolation domain

## Abstract

Non-ribosomal peptide synthetases (NRPSs) are large multienzyme machineries. They synthesize numerous important natural products starting from amino acids. For peptide synthesis functionally specialized NRPS modules interact in a defined manner. Individual modules are either located on a single or on multiple different polypeptide chains. The “peptide-antimicrobial-*Xenorhabdus*” (PAX) peptide producing NRPS PaxS from *Xenorhabdus* bacteria consists of the three proteins PaxA, PaxB and PaxC. Different docking domains (DDs) located at the N-termini of PaxB and PaxC and at the C-termini of PaxA and BaxB mediate specific non-covalent interactions between them. The N-terminal docking domains precede condensation domains while the C-terminal docking domains follow thiolation domains. The binding specificity of individual DDs is important for the correct assembly of multi-protein NRPS systems. In many multi-protein NRPS systems the docking domains are sufficient to mediate the necessary interactions between individual protein chains. However, it remains unclear if this is a general feature for all types of structurally different docking domains or if the neighboring domains in some cases support the function of the docking domains. Here, we report the ^1^H, ^13^C and ^15^ N NMR resonance assignments for a C-terminal di-domain construct containing a thiolation (T) domain followed by a C-terminal docking domain (^C^DD) from PaxA and for its binding partner – the N-terminal docking domain (^N^DD) from PaxB from the Gram-negative entomopathogenic bacterium *Xenorhabdus cabanillasii* JM26 in their free states and for a 1:1 complex formed by the two proteins. These NMR resonance assignments will facilitate further structural and dynamic studies of this protein complex.

## Biological context

Non-ribosomal peptide synthases (NRPS) use amino acids as building blocks for the synthesis of complex natural products. Each amino acid is incorporated into the final product by an individual specialized functional module of the NRPS. In turn, each individual module contains a number of enzymatically distinct catalytic domains. A typical NRPS elongation module contains at least an adenylation (A), a thiolation (T) and a condensation (C) domain. The adenylation (A) domain uses ATP to activate a specific amino acid as aminoacyl adenylate and then transfers the activated amino acid to the neighbouring thiolation (T) domain. The T domain in its *holo* form contains a phosphopantetheinyl (Ppant) moiety derived from coenzyme A covalently bound to a conserved serine side chain. The activated amino acid reacts with the terminal thiol group of the Ppant moiety to form a thioester. The T domain is followed by a condensation domain (C) which catalyses the formation of peptide bonds between the amino acid bound to the T domain of its own module and an amino acid or a peptide chain bound to the T domain of the downstream module. Additional domains with enzymatic activities such as methylation or epimerization can be included in individual modules and may further modify the peptide product. Finally, the nascent peptide is released by a thioesterase (TE) domain localized at the C-terminus of a specialized termination module. TE domains can release either linear, cyclic or branched cyclic peptides (Süssmuth and Mainz 2017).

The individual functional modules necessary for the stepwise incorporation of each amino acid into the final product can be located either on a single long protein chain or on multiple proteins. When all modules are arranged on a single protein chain their linear order directly predicts (Mootz et al. 2002) the order of amino acid building blocks in the final product. In multi-protein NRPS systems specific non-covalent interactions between the individual protein chains determine the functional assembly of the NRPS complex and thereby the order of amino acids in the synthesized peptide. Hahn and Stachelhaus (Hahn and Stachelhaus 2004) were first in identifying short amino acid sequences at the N- and C-termini of the individual protein chains in a multi-protein NRPS that were capable of mediating specific noncovalent interactions between these protein chains. They referred to these sequences as “communication-mediating (COM) domains”. Functionally similar structural elements, which were named “docking domains (DD)” (Broadhurst et al. 2003), were identified in polyketide synthase (PKS) complexes which possess a multi-modular architecture similar to NRPS systems. The term “docking domain” is now more commonly used for domains that enable non-covalent specific interactions between individual protein chains in both PKS and NRPS complexes. Multiple structurally diverse families of DD architectures have been described so far (Broadhurst et al. 2003; Buchholz et al. 2009; Dorival et al. 2016; Hacker et al. 2018; Watzel et al. 2020; Whicher et al. 2013). Many DD structural families are dominated by α-helical secondary structure elements (Buchholz et al. 2009; Watzel et al. 2020; Whicher et al. 2013). The binding affinities even for specific interactions between DDs are relatively weak with typical dissociation constants for the DD complexes in the range between 5 µM and 25 µM (Dorival et al. 2016; Hacker et al. 2018; Watzel et al. 2020; Whicher et al. 2013). Nevertheless, for many types of DD interactions it has been experimentally demonstrated that the DDs act independently from the other functional domains in an NRPS or PKS in order to mediate the non-covalent interactions between protein chains needed for the functional assembly of functional megasynthase complexes (Dorival et al. 2016; Hacker et al. 2018). However, it is not yet clear if this is true for all types of docking domains.

The PaxS NRPS found in Gram-negative entomopathogenic bacteria from the genus *Xenorhabdus* is a prototypical example for a multi-protein assembly line. It consists of the three proteins PaxA, PaxB and PaxC. The three proteins interact with each other non-covalently in a unidirectional manner where PaxA is bound by PaxB and PaxB is bound by PaxC. The DD pair mediating the specific interaction between the C-terminus of PaxB and the N-terminus of PaxC has been characterized functionally, structurally and biophysically in previous work (Kegler and Bode 2020; Watzel et al. 2020). A structural basis for the specific interaction between the C-terminus of PaxA and the N-terminus of PaxB has not yet been established. Notably, the putative C-terminal docking domain (^C^DD) in PaxA was predicted to be very short in comparison to other structurally characterized types of docking domains. Thus, in this case the T domain directly preceding the ^C^DD of PaxA might play a supporting role in establishing a specific non-covalent interaction with the N-terminal docking domain (^N^DD) of PaxB. As a prerequisite for investigating a putative role of the T domain in this DD interaction and for establishing a structural basis for the specific interaction between PaxA and PaxB in the PaxS NRPS from *Xenorhabdus cabanillasii* JM26 we present here the NMR resonance assignments for a PaxA T_1_-^C^DD di-domain construct and for the PaxB ^N^DD in their free states and in a 1:1 non-covalent complex. The T_1_-^C^DD (amino acids 981–1084 of PaxA) comprises 104 amino acids (12 kDa), whereas the ^N^DD (amino acids 1–30 of PaxB) contains only 30 amino acids (3.6 kDa).

## Methods and experiments

### Cloning, expression and purification

The DNA coding sequences for PaxA T_1_-^C^DD and PaxB ^N^DD were generated by PCR amplification using the genomic DNA from *Xenorhabdus cabanillasii* JM26 as the template. All protein sequences referred to in this work are based on the UniProt Archive (UniParc) entries for PaxA (UPI0003E57C57) and PaxB (UPI000C04EDD1) based on a genome assembly for *Xenorhabdus cabanillasii* JM26 produced in our group (NCBI: ASM263290v1; GenBank: NJGH00000000 (Tobias et al. 2017)). For protein concentration measurements a codon for a tyrosine residue was added downstream of the PaxB ^N^DD coding sequence via the used primer. The inserts were cloned into a modified pET-11a vector (Hacker et al. 2018) containing the sequence for an N-terminal hexahistidine (His_6_)-tag followed by a SUMO (SMT3)-tag which also functions as a cleavage site for the ULP1 protease (Malakhov et al. 2004). The PaxA T_1_-^C^DD was expressed in its *apo* form by using *Escherichia coli* BL21(DE3)Δ*entD* (Owen et al. 2012) cells preventing post-translational modification of the T domain by the covalent addition of a phosphopantetheinyl moiety, whereas the PaxB ^N^DD was expressed in *E. coli* BL21(DE3) Gold (Agilent Technologies/Stratagene) cells.

Protein expression was induced at OD_600_ ~ 0.7 with 1 mM IPTG at 20 °C for ~ 18 h in media supplemented with ampicillin (100 µg/ml). The expression of uniformly ^15^ N- and ^15^ N,^13^C-labelled proteins was achieved by using M9 minimal medium supplemented with 1 g/l ^15^NH_4_Cl or 1 g/l ^15^NH_4_Cl and 2.5 g/l ^13^C_6_-*D*-glucose (Cambridge Isotope Laboratories). For the stereospecific NMR assignment of γ^1/2^ and δ^1/2^ methyl groups of valine and leucine, the PaxA T_1_-^C^DD as well as the PaxB ^N^DD were expressed as fractionally ^13^C-labelled proteins in M9 minimal medium containing a mixture of 0.25 g L^−1 13^C_6_-*D*-glucose and 2.25 g L^−1^ unlabeled glucose as the sole carbon source (Neri et al. 1989).

Cell lysis of *E. coli* cells was accomplished by sonication in a buffer containing 50 mM sodium phosphate, pH 8.0, 300 mM NaCl, 1 mM EDTA, 10 mM MgCl_2_, 2 mM β-mercaptoethanol, Benzonase (Merck) and cOmplete protease inhibitor (Roche). The cell debris was cleared from the lysate by centrifugation (8000 × *g*, 4 °C, 30 min) and the supernatant was run through a HisTrap HP column (GE Healthcare). With the help of a recombinantly produced His_6_-tagged ULP1 protease the His_6_-tagged SUMO (SMT3)-tag was cleaved off from the recombinant fusion proteins, leading to native target protein sequences. Both His_6_-tagged ULP1 and the SUMO-tag were separated from the protein of interest by a second immobilized metal ion affinity chromatography using a HisTrap HP column, followed by gel filtration chromatography with a HiPrep 16/60 Sephacryl S-100 high resolution column (GE Healthcare). For NMR measurements, the samples of the individual proteins (300 µM protein concentration) and the PaxA T_1_-^C^DD:PaxB ^N^DD/PaxB ^N^DD:PaxA T_1_-^C^DD complexes (300 µM protein A concentration: 360 µM protein B concentration) were prepared in a buffer containing 50 mM sodium phosphate, pH 6.5, 100 mM NaCl, 2 mM β-mercaptoethanol and 5% (v/v) D_2_O.

### NMR spectroscopy

NMR experiments were recorded at 293 K on Bruker AVANCE III HD 600, 700 and 800 MHz spectrometers, each of them equipped with 5 mm cryogenic triple resonance probes. ^1^H chemical shifts were internally referenced to DSS, whereas the heteronuclear ^13^C and ^15^ N chemical shifts were indirectly referenced with the appropriate conversion factors (Markley et al. 1998). Spectra were processed using TOPSPIN 3.6.2 (Bruker) and analysed with CARA (Keller 2004). The secondary structure of the unbound and bound PaxA T_1_-^C^DD and PaxB ^N^DD was derived from TALOS-N (Shen and Bax 2013) based on the chemical shift assignments.

For the backbone assignment of the free PaxA T_1_-^C^DD a uniformly ^13^C,^15^ N-labelled sample was used and the following triple resonance experiments were recorded: HNCO, HNCA, HNCACB, HBHA(CO)NH, CBCA(CO)NH (Sattler 1999). The backbone and side chain assignments of the unbound ^13^C,^15^ N-labelled PaxB ^N^DD were derived from 3D HNCO, HNCACB, HBHA(CO)NH, H(CCO)NH (mixing time 12 ms) and (H)C(CO)NH (mixing time 12 ms) experiments (Sattler 1999). The backbone resonances of ^13^C,^15^ N-labelled PaxA T_1_-^C^DD/PaxB ^N^DD in complex with a 1.2-fold excess of unlabelled PaxB ^N^DD/PaxA T_1_-^C^DD were assigned on the basis of 3D HNCO, HNCA, HNCACB, and CBCA(CO)NH experiments (Sattler 1999). Side chain assignments were obtained from 3D HBHA(CO)NH, H(C)CH-/(H)CCH-TOCSY (mixing times 12 ms) (Sattler 1999) and H(C)CH-COSY (Bax et al. 1990) experiments. Stereospecific assignments of valine γ^1/2^ and leucine δ^1/2^ methyl groups of the bound PaxA T_1_-^C^DD and free and bound PaxB ^N^DD were determined in ^1^H,^13^C-HSQC experiments with a resolution in the ^13^C dimension of ~ 28 Hz, ~ 26 Hz and ~ 23 Hz, respectively. This allowed the unambiguous discrimination between the signals for the γ^2^/ δ^2^ CH_3_ groups of valine and leucine which appear as singlets in the ^13^C-dimension and the signals of the γ^1^/ δ^1^ CH_3_ groups which appear as doublets separated by the ^1^*J*_13C,13C_ coupling constant of ~ 33 Hz (Neri et al. 1989).

### Assignment and data deposition

**Fig. 1:**
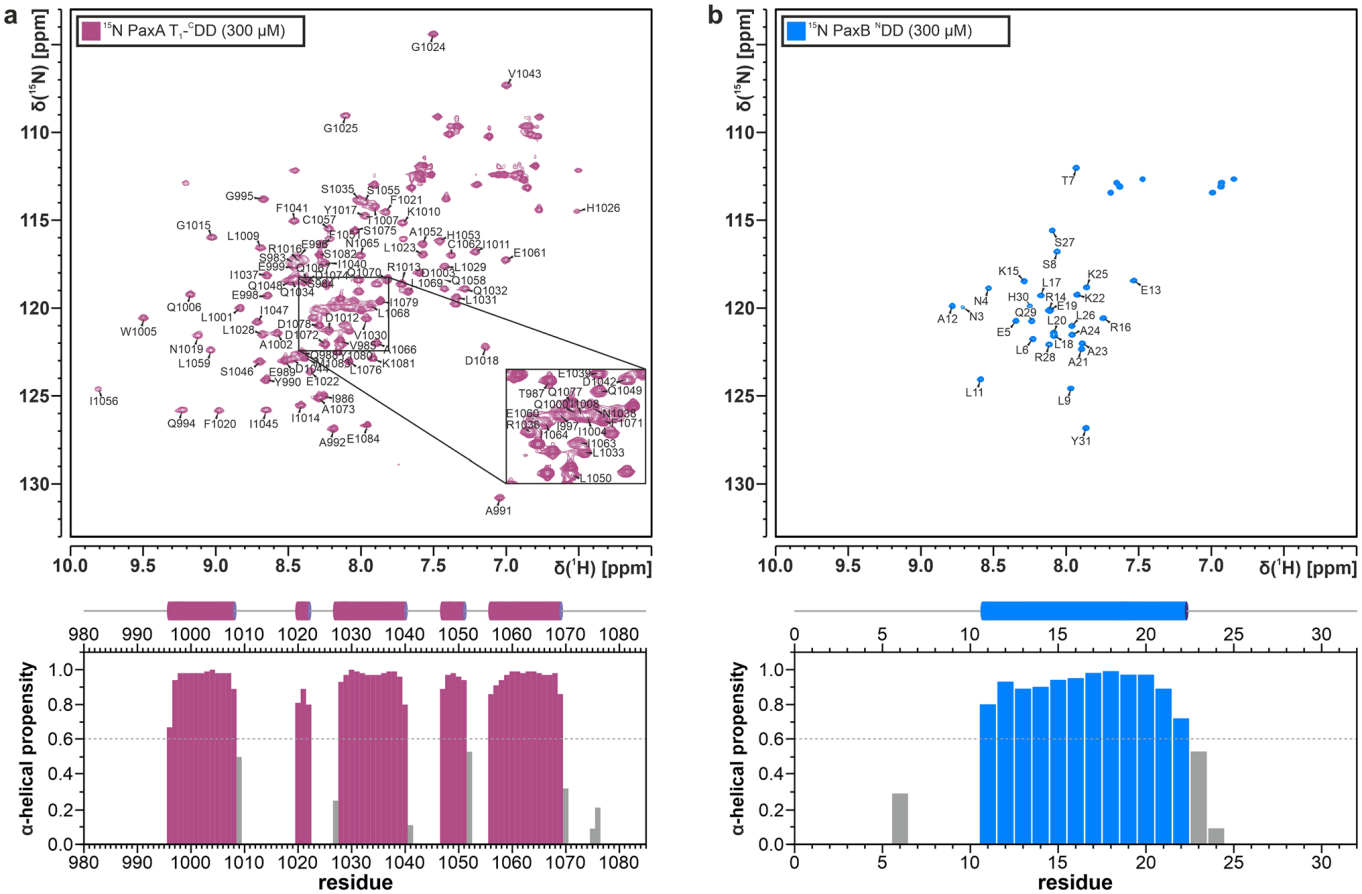
(**a**, top) ^1^H,^15^ N-HSQC spectra and assigned backbone amide signals for the free PaxA T_1_-^C^DD and (**b**, top) the free PaxB ^N^DD. The spectra were recorded at 293 K on a 600 MHz Bruker Avance III spectrometer. The assigned NMR signals are labelled with their respective residue names and numbers and the central spectral region of the PaxA T_1_-^C^DD with increased peak overlap is enlarged for a better overview (box). (**a**, **b**, bottom) TALOS-N based secondary structure analysis

The protein construct PaxA T_1_-^C^DD contains amino acids 981 to 1084 of the PaxA protein from *Xenorhabdus cabanillasii* JM26. It has a molecular weight of 12 kDa and consists of 104 amino acid residues. Most of the backbone amid signals are well dispersed in the ^1^H,^15^ N-HSQC spectrum of the unbound PaxA T_1_-^C^DD as is typical for a well-folded protein. The ^1^H,^15^ N-HSQC is expected to contain 101 backbone amide signals since there is no observable amide signal for the N-terminal residue D981 and there are two proline residues (P993, P1054) present in the sequence. However, only 99 backbone amide signals were observed and assigned (99/101, 98.0%; Fig. 1a, top). No backbone amide signals were detectable for residues H982 and S1027 most likely due to fast exchange of the respective amide protons with the solvent or due to conformational exchange. S1027 is the residue that would be posttranslationally modified by addition of the Ppant-arm in the native environment. Furthermore, 99% of all Cα (103/104), 98% of all Cβ (98/100), 95% of all CO (99/104) and Hα (99/104) and 94% of all Hβ (94/100) chemical shifts were assigned. The backbone chemical shifts were used to derive the putative secondary structure of the free PaxA T_1_-^C^DD. Based on these chemical shifts TALOS-N (Shen and Bax 2013) identified five amino acid stretches with high α-helical propensity (Fig. 1a, bottom). This secondary structure fits well to that of previously described T domain structures, which typically feature a four-helix bundle (Weber et al. 2000). Compared to the canonical four-helix bundle T domain fold the PaxA T_1_ domain contains an additional very short (3 residues) α-helix in the linker region between the canonical helices α1 and α2. Such an additional helix is also present at this position in some other carrier protein structures (Lohman et al. 2014). According to TALOS-N the C-terminus including the predicted ^C^DD of the PaxA T_1_-^C^DD construct is unstructured.

The PaxB ^N^DD construct contains amino acids 1–30 of the PaxB protein followed by a non-native tyrosine at the C-terminus and has a molecular weight of 3.6 kDa. Surprisingly, the backbone amide signals of the free PaxB ^N^DD are well dispersed in the ^1^H,^15^ N-HSQC spectrum (Fig. 1b, top) indicating a folded protein. Overall, 27 backbone amide signals were assigned since the sequence contains one proline residue (P10) and no backbone amide signals were observable for M1, N2 and the non-native Y31. Additionally, 93% of all CO (28/30) and all Cα (30/30), Cβ (30/30), Hα (30/30) and Hβ (30/30) chemical shifts were assigned. The overall side chain assignments were completed to 99.2% for aliphatic protons and to 99.0% for aliphatic carbons. No aromatic side chain NMR signals were assigned since the PaxB ^N^DD contains only a single native aromatic residue (H30) and the non-native Y31. The TALOS-N analysis of the secondary structure suggests the presence of a single continuous α-helix including residues L11 to K22 in the free PaxB ^N^DD (Fig. 1b, bottom). The ^13^Cγ and ^13^Cβ chemical shifts of proline P10 are in agreement with a *trans* conformation for this residue (Schubert et al. 2002). In addition, all valine γ^1/2^ and leucine δ^1/2^ methyl groups of the unbound PaxB ^N^DD were stereospecifically assigned.

**Fig. 2:**
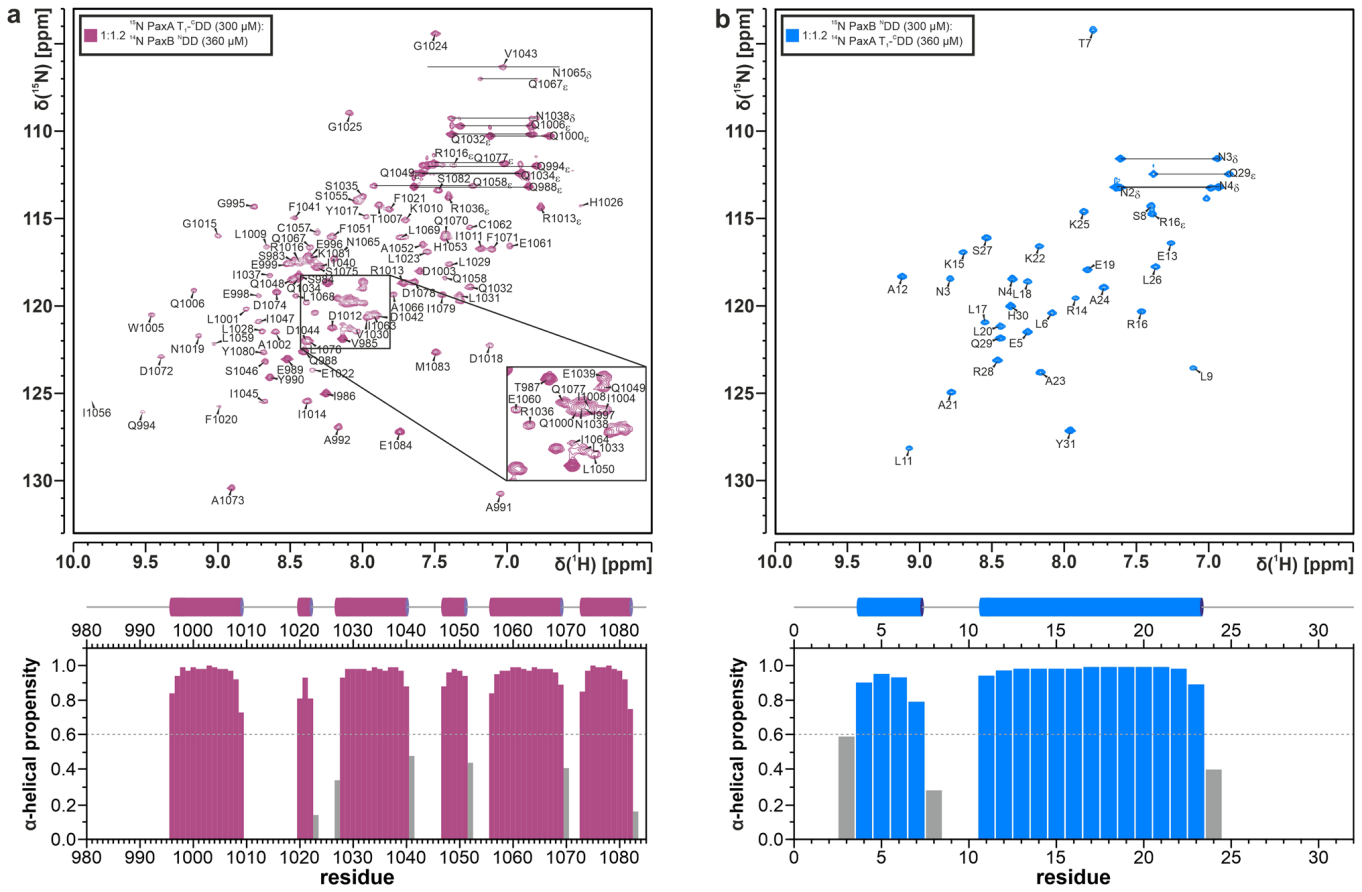
(**a**, top) ^1^H,^15^ N-HSQC spectra and assigned backbone amide signals for the bound PaxA T_1_-^C^DD and (**b**, top) the bound PaxB ^N^DD. The spectra were recorded at 293 K on a 600 MHz Bruker Avance III spectrometer. The assigned NMR signals are labelled with their respective residue names and numbers. The central spectral region of the PaxA T_1_-^C^DD with increased peak overlap is enlarged for a better overview (box). Assigned sidechain NH_2_ signals are indicated by horizontal bars and labelled with the respective residue names and numbers. The labelled ϵ imino groups of R1013, R1016 and R1036 in the PaxA T_1_-^C^DD and of R16 in the PaxB ^N^DD are folded into the spectrum. (**a**, **b**, bottom) TALOS-N based secondary structure analysis

PaxA T_1_-^C^DD and PaxB ^N^DD form a stable 1:1 complex according to analytical gel filtration and NMR titration experiments which is in slow exchange on the NMR time scale. Assignments for both proteins in their bound states were made using samples containing one binding partner in ^13^C,^15^ N-labelled form and a 1.2-fold excess of the unlabelled binding partner. The backbone resonances of PaxA T_1_-^C^DD bound to the PaxB ^N^DD could be assigned with the same degree of completeness as for the free protein. All expected backbone amide signals except those for H982 and S1027 (99/101, 98.0%; Fig. 2a, top) and all Cα (104/104), 99% of all Cβ (99/100), 95% of all CO (99/104), all Hα (104/104) and 99% of all Hβ (99/100) chemical shifts were assigned. The overall side chain assignments were completed to 98.7% for the remaining aliphatic protons and to 98.2% for aliphatic carbons. All valine γ^1/2^ and leucine δ^1/2^ methyl groups were stereospecifically assigned. In addition, 56.3% of all aromatic side chain proton-bound carbon and carbon-bound proton signals were assigned.

The analysis of the backbone chemical shifts of the PaxA T_1_-^C^DD with TALOS-N in its bound state suggests that its secondary structure is very similar to that in its free state but that there is now an additional α-helix present at the very C-terminus suggesting that the predicted docking domain becomes structured upon binding (Fig. 2a, bottom).

The backbone assignment of the bound PaxB ^N^DD (Fig. 2b, top) includes 27 amide backbone signals, 93% of all CO (28/30) and all Cα (30/30), Cβ (30/30), Hα (30/30) and Hβ (30/30) chemical shifts. The overall side chain assignments were completed to 99.2% for aliphatic protons and to 99.0% for aliphatic carbons. All valine γ^1/2^ and leucine δ^1/2^ methyl groups were stereospecifically assigned. In addition, 50% of all aromatic side chain proton-bound carbon and carbon-bound proton signals were assigned. The TALOS-N derived secondary structure for the bound PaxB ^N^DD indicates the presence of two α-helices (Fig. 2b, bottom). The proline residue P10 remains in the *trans* conformation in the bound state according to its ^13^Cγ and ^13^Cβ chemical shifts (Schubert et al. 2002).

The assigned chemical shifts have been deposited in the BMRB under the accession numbers 50,594, 34,576 and 34,575 for the free PaxA T_1_-^C^DD construct, the free PaxB ^N^DD and the complex, respectively.
